# Multimodality Treatment in Metastatic Gastric Cancer: Working Together to Tailor the Continuum of Care

**DOI:** 10.3390/jcm10235492

**Published:** 2021-11-24

**Authors:** Angelica Petrillo

**Affiliations:** Medical Oncology Unit, Ospedale del Mare, 80147 Naples, Italy; angelic.petrillo@gmail.com

Gastric cancer (GC) represents one of the most frequent and lethal tumors worldwide today, finding itself in fifth place in terms of incidence and third in terms of mortality [1]. Surgery remains the only curative treatment for localized tumors, but only 20% of patients are suitable for surgery due to the lack of specific symptoms and the late diagnosis, especially in Western countries [2]. Additionally, even in patients who receive curative treatment, the rates of locoregional relapse and distant metastases remain high [3]. Palliative chemotherapy is the principal treatment in cases of metastatic disease; however, the prognosis of patients receiving chemotherapy is still poor. In this context, one of the most important conceptual achievements of the last few decades in the field of metastatic GC is represented by the “continuum of care”, meaning the possibility to treat patients with multiple subsequent lines of therapy in order to obtain longer survival. In fact, almost 40% of patients receiving first-line treatment for metastatic disease maintain a good performance status after progression; they are able to receive a second and even a third line of treatment. Additionally, there is an unmet need for a better understanding of genetic alterations and prognostic and predictive factors in metastatic GC that could be useful when choosing the best-tailored therapy for each patient [4].

Therefore, a multidisciplinary evaluation is crucial in order to individualize treatment for patients with metastatic GC—according to the concept of precision medicine—and to define the right sequence of treatment from the diagnosis—according to the concept of “continuum of care”. Taking all these facts into account, the aim of this Special Issue was to focus on the results and problems of multimodality treatment in metastatic GC, the search for prognostic and predictive factors, and the evaluation of novel strategies for individualized treatment. 

In this regard, the management of patients with oligometastatic GC is challenging. In particular, the treatment for patients with only liver metastases is changing and the latest advances in the research in this field suggest a potential role for multimodality treatment, including curative surgery. In this Special Issue, Marte, G. et al. [5] provided a systematic review and metanalysis of 40 studies with the aim of evaluating the efficacy of hepatectomy for metastatic GC with liver metastases. The authors showed that an approach consisting of the resection of the primary tumor alongside liver metastases by hepatectomy is feasible in this population and provides benefits in terms of long-term survival. However, the clear definition of oligometastatic disease and the role of multimodality treatment for these patients are still a matter of debate; clinical trials are ongoing in this setting. Additionally, a strict selection of patients that could benefit from curative surgery is mandatory and requires a multidisciplinary evaluation.

In the field of metastatic GC today, it is still unclear how the metastatic sites may affect the prognosis and little evidence exists regarding the impact of rare metastatic locations (e.g., lung, bone and brain). Therefore, a narrative review regarding the role of bone metastases in GC and their potential implication in treatment choice was also included in this Special Issue [6]. 

A nutritional assessment is crucial in the multidisciplinary evaluation of metastatic GC patients. However, few data about the link between nutritional status and survival are available in this field and the role of sarcopenia in metastatic GC is controversial today. In this Special Issue, Rimini, M. et al. [7] evaluated the prognostic role of tissue modifications (assessed using computed tomography) during treatment in 40 metastatic GC patients and the benefit of a scheduled nutritional assessment in this setting. Interestingly, the authors showed that an early skeletal muscle mass depletion >10% in the first months of treatment significantly influenced the overall survival (*p* = 0.0023). Additionally, Catanese, S. et al. [8] investigated the role of baseline computed-tomography-evaluated body composition in predicting the outcome and toxicity of first-line therapy in 78 advanced GC patients. In this paper, even if sarcopenia failed to show an association with the outcomes, skeletal muscle mass depletion was linked to the development of high-grade neutropenia (*p* = 0.048) and mucositis (*p* = 0.054). On the other hand, the fat distribution (visceral versus subcutaneous) exhibits a robust impact on survival.

However, although GC has been considered as a single entity for a long time, nowadays it is acknowledged that it represents a heterogeneous disease, deserving to be treated according to the own peculiarities of each subtype. Based on this background, several molecular classifications have been developed over the last few decades in an attempt to select molecular alterations, which might act as a driver for each subtype [4]. 

In this context, the application of sequencing has led to the identification of aberrant druggable pathways and somatic mutations within therapeutically relevant genes in GC. However, since the majority of those evaluations use formalin-fixed paraffin-embedded (FFPE) samples, an assessment of the concordance between comprehensive exome-wide sequencing data from archival FFPE samples would be beneficial in order to find some potential biomarker in GC. In this regard, Chong, I.Y. et al. [9] reported in this Special Issue the analysis of whole-exome sequencing data from 16 matched fresh-frozen and FFPE gastro-esophageal tumors (N = 32) with the aim of defining the mutational concordance. The authors found a high median mutational concordance (97%) between fresh-frozen and FFPE gastro-esophageal tumor-derived exomes, suggesting that comprehensive genomic data can be generated from the exome sequencing of selected DNA samples extracted from archival FFPE samples.

The molecular peculiarities of GC subtypes can influence the treatment choice in the metastatic setting. Based on that, Gambardella, V. et al. [10] provided a comprehensive overview of the role of precision medicine in metastatic GC. In particular, the authors described the novel pathways implicated in GC and the state of the art of target therapies in those tumors, according to molecular classifications and alterations. Even if the road toward a personalized approach requires further studies, this paper underlined the importance of molecular selection to use tailored treatments for metastatic GC. 

In this scenario, the development of immunotherapy could also represent a promising strategy in a selected population affected by metastatic GC. In this Special Issue, Ghidini, M. et al. [11] conducted an update of the predictive biomarkers of response to immunotherapy in GC. Additionally, the authors provided an overview regarding the translational meaning of those findings in the practice, alongside descriptions of the landmark and ongoing clinical trials in this field.

Lastly, one of the most important frontiers in the GC scenario is represented by the development and use of liquid biopsy, both in localized and metastatic disease. In this Special Issue, Lengyel, C.G. et al. [12] summarized the state of the art and future application of liquid biopsy in GC, showing that, although preliminary results are promising, more research is required to obtain better insights into the molecular mechanisms, as well as to validate and standardize the methods for liquid biopsy. 

In conclusion, the papers collected in this Special Issue highlight that the treatment of metastatic GC is challenging. A careful and comprehensive evaluation of these patients by a multidisciplinary team in dedicated and high-volume centers is crucial in order to improve the outcomes. The multidisciplinary evaluation should include a nutritional assessment, since sarcopenia and fat distributions might affect the prognosis and the rate of drug toxicities, as well as the evaluation of metastases’ distribution and the definition of oligometastatic disease, which is at the forefront of metastatic GC research today. In this context, the role of surgery as part of a multimodal strategy for metastatic disease is improving. We should consider that GC is a very heterogeneous disease; therefore, we should attempt to treat each patient according to the tumor and patient characteristics, which lead to the choice of a unique and personalized treatment journey, based on the milestone concepts of precision medicine and a continuum of care. 

However, the journey to discover the molecular mechanisms that control GC behavior has just started and further investigation is still required. Thus, working together as a multidisciplinary team including different professional figures, such as oncologists, nutritionists, surgeons, pathologists, radiotherapists, gastroenterologists, etc., is the only way to achieve proper patient care in the complex and evolving landscape of metastatic GC.
